# Implementation of Resonant Electric Based Metamaterials for Electromagnetic Wave Manipulation at Microwave Frequencies

**DOI:** 10.3390/s21248452

**Published:** 2021-12-18

**Authors:** Stylianos D. Assimonis, Sandhya Chandravanshi, Okan Yurduseven, Dmitry Zelenchuk, Oleksandr Malyuskin, Muhammad Ali Babar Abbasi, Vincent Fusco, Simon L. Cotton

**Affiliations:** Centre for Wireless Innovation (CWI), Institute of Electronics Communications and Information Technology, Queen’s University Belfast, Belfast BT3 9DT, Northern Ireland, UK; S.Chandravanshi@qub.ac.uk (S.C.); Okan.Yurduseven@qub.ac.uk (O.Y.); D.Zelenchuk@qub.ac.uk (D.Z.); O.Malyuskin@qub.ac.uk (O.M.); M.Abbasi@qub.ac.uk (M.A.B.A.); V.Fusco@ecit.qub.ac.uk (V.F.); Simon.Cotton@qub.ac.uk (S.L.C.)

**Keywords:** compressive sensing, direction of arrival estimation, energy harvesting, metamaterial, metasurface, microwave absorption, reflecting surface, wireless power transfer

## Abstract

In this paper, we present the application of a resonant electric based metamaterial element and its two-dimensional metasurface implementation for a variety of emerging wireless applications. Metasurface apertures developed in this work are synthesized using sub-wavelength sampled resonant electric-based unit-cell structures and can achieve electromagnetic wave manipulation at microwave frequencies. The presented surfaces are implemented in a variety of forms, from absorption surfaces for energy harvesting and wireless power transfer to wave-chaotic surfaces for compressive sensing based single-pixel direction of arrival estimation and reflecting surfaces. It is shown that the resonant electric-synthesized metasurface concept offers a significant potential for these applications with high fidelity absorption, transmission and reflection characteristics within the microwave frequency spectrum.

## 1. Introduction

Over the past decade, researchers have increasingly prioritized their focus on the field of metamaterials [1,2]. Essentially, they are inherently inhomogeneous structures that consist of periodically repeated unit-cells, usually arranged on square lattices in two or three dimensional structures. Their properties mainly arise from their geometric details rather than their constituting material properties. Metamaterial unit-cell structures usually consist of various combinations of an electric (e.g., vertical wire) and magnetic (e.g., wire loop) resonator that couple separately to electric and magnetic fields. The interest for studying metamaterials mainly arises from their use in potential applications. Some of these applications include emitters [3,4], sensors [5], spatial light modulators [6], infrared (IR) camouflage [7,8], thermo-photovoltaics [9] and wireless communication [10,11], etc. Moreover, they are easily fabricated to interact with electromagnetic (EM) waves in the microwave regime. Essentially, initial concepts have been realized on low-cost FR-4 substrates by means of standard photo-lithographic techniques and proven to be very sub-wavelength, with a thickness of around λ/30 at their resonance frequency.

A particular section, the metamaterial perfect absorbers (MPAs), has attracted significant attention due to their ability to offer near unity absorption of electromagnetic waves [12,13]. As metamaterial absorbers are thinner than the traditional microwave absorbers [14], due to diffraction limit restrictions, they are perfect candidates to replace them as absorbing elements in scientific and technical applications. In fact, the novel idea proposed by Landy et al. [12] has led the prime focus in the analysis and fabrication of absorbing devices that operate at microwave, terahertz, and even visible frequencies [15]. Another technique used in the design of absorbing surfaces is the utilization of electromagnetic band-gaps (EBGs) [16,17], which usually leads to thicker unit-cells with dimensions comparable to the wavelength.

As modern practical applications continue to raise requirements, such as larger bandwidths or multiple bands of operation, significant research has been initiated to improve the overall behavior of the current generation of absorbers [18,19,20,21,22]. One of the first proposed multi-band approaches in the microwave region used four identical electric ring resonators (ERRs) in their unit-cell and achieved a dual resonance by rotating two of the structures by 90 degrees [23]. Other studies utilized the same idea to achieve extended-banded responses in the near-infrared (NIR) regime [7,13]. In an example of a multi-band MPA, some studies used the metamaterials scalability property of multiple electric inductor-capacitors in their unit-cell to achieve a multi-absorption response. They were able to do this with two and three separately resonating structures. With these techniques it is possible to create multiple resonance bands or, if the resonances are close enough in frequency to one another, to create a single broadband peak [24].

The two-dimensional composition of metamaterial structures to form flat-panel metasurface-based apertures has recently received significant attention [3,6,25,26]. An advantage of metasurface-based aperture modalities is that they can manipulate the EM waves in a holographic manner [4]. The holographic control of EM waves has several benefits over conventional phased array based approaches, such as eliminating the need for hardware-intense phase shifting circuits, simplified physical hardware layer and reduced power consumption.

The goal of this paper is to present the promising potential of resonant electric (ELC) based metasurface apertures [27] for a variety of emerging applications at microwave frequencies. In this context, we first propose a new polarization insensitive and wide-angle metamaterial harvester, consisting of conventional ELC metamaterial structures. In this concept, the design procedure is considered of low complexity since it is focused on the unit-cell and not on the periodic structure, by incorporating an equivalent rectangular unit-cell. Next, we test the metasurface concept for wireless power transfer (WPT) with a dedicated rectifier unit, designed specifically for the presented WPT framework. Following this, we present the concept of compressive sensing based direction of arrival (DoA) estimation facilitated by wave-chaotic metasurface antennas synthesized using complementary ELC unit-cells. In this study, we show that it is possible to retrieve the DoA information of an arbitrary number of far-field sources using a single-channel architecture facilitated by physical layer compression. Finally, we present the importance of the pathloss concept in designing these metasurface apertures.

## 2. Methods and Results

### 2.1. Wireless Power Transfer Using Metasurfaces

The basic role of EM absorbers in various applications is to eliminate the unwanted electromagnetic energy which arises as a result of scattering. The term absorbing surface is used to describe those geometries that have the ability to reduce effectively the transmission and reflection of the incident radiation. The most important parameter for metamaterial absorbers is the absorption, which is interpreted as the percentage of incident energy absorbed by the material. For the efficient design of metamaterial absorbers, it is necessary to minimize both reflection and transmission of incident waves. Such performance can be achieved due to the complex interactions of the metamaterial with the impinging radiation. This stems from the definition of absorption, obtained in terms of the *S*-parameters as:

(1) A=1−T−R,

where $T={\left|S_{21}\right|}^{2}$ is the transmission and $R=|S_{11}{|}^{2}$ the reflection of the device. For a slab of length *d*, coefficients *R* and *T* can be derived directly from simulation or measurement of its *S*-parameters.

In a metamaterial harvester (MH), a fraction of the absorbed power dissipates in the dielectric and metallic parts of the geometry and the rest of the power is delivered to the RF-to-dc rectifier. The latter is usually simulated by a load, which represents the input impedance of the rectification system. The efficiency of a MH is defined as the ratio of the delivered power to the RF-to-dc rectifier $P_{\ell }$ to the incident power $P_{i}$, i.e.,

(2) $$\eta_{\ell }=\frac{P_{\ell }}{P_{i}}$$

Thus, absorption is always greater or at least equal to the MH’s efficiency, i.e., $A\geq \eta_{l}$.

A logical extension of the MHs lies in the field of wireless power transfer (WPT). Research into WPT has intensified significantly over the last decade [28], with many new applications emerging and necessitating this technology, a prime example being unmanned aerial vehicles (UAVs) [29,30]. These applications require mid- to high-power range WPT with power levels of 20 dBm and above being delivered to the end user. The metasurface energy harvesting in such a case possesses all the advantages of low power harvesting mentioned in the sections above, and such planar systems are increasingly attractive for WPT [31,32,33]. Yet as the power level increases, the rectifying elements require matching circuitry to increase conversion efficiency [34].

**Figure 1:**
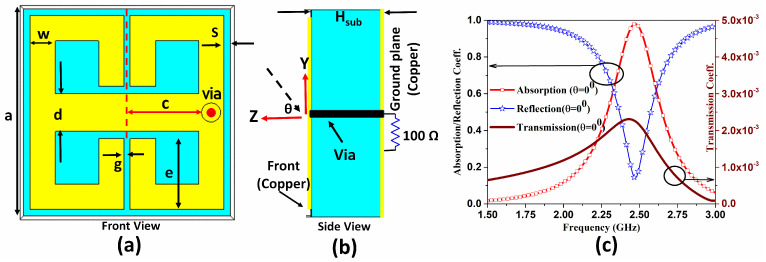
The designed unit-cell of the proposed structure for a WPT application (**a**) Front view of unit-cell (**b**) side view of the unit-cell with air as a substrate, (**c**) the simulated reflection, transmission and absorption of the unit-cell using CST software.

The proposed design in this work consists of a compact unit-cell, directly adopted from [20]. The front view of the unit-cell is shown in Figure 1a. The unit-cell is optimized for a single band of frequency at 2.45 GHz. The air substrate of thickness $H_{sub}$ is considered between the metallic ground plane and front end metallic resonating structure. When a plane wave impinges on the surface with some angle (θ) from the normal to the surface, an electric current is induced and flows through the 1.5 mm thick via to the 100 Ω resistive load behind the ground plane, as shown in Figure 1b.

The unit-cell is optimised to absorb a linearly polarised plane wave and as shown in Figure 1c, the reflection and transmission (right axis) are very low at the design frequency, resulting in an absorption efficiency of 98%. The parameters of the unit-cell are given in Table 1. At final stage of the design, a dedicated rectifier will replace the resistive load, which at present mimics the input impedance of a rectification system [20].

In order to ensure polarisation-insensitive behavior of the structure and taking advantage of the subwavelength size of the unit-cell we combine adjacent cells into a 2 × 2 supercell, similar to [13].

**Figure 2:**
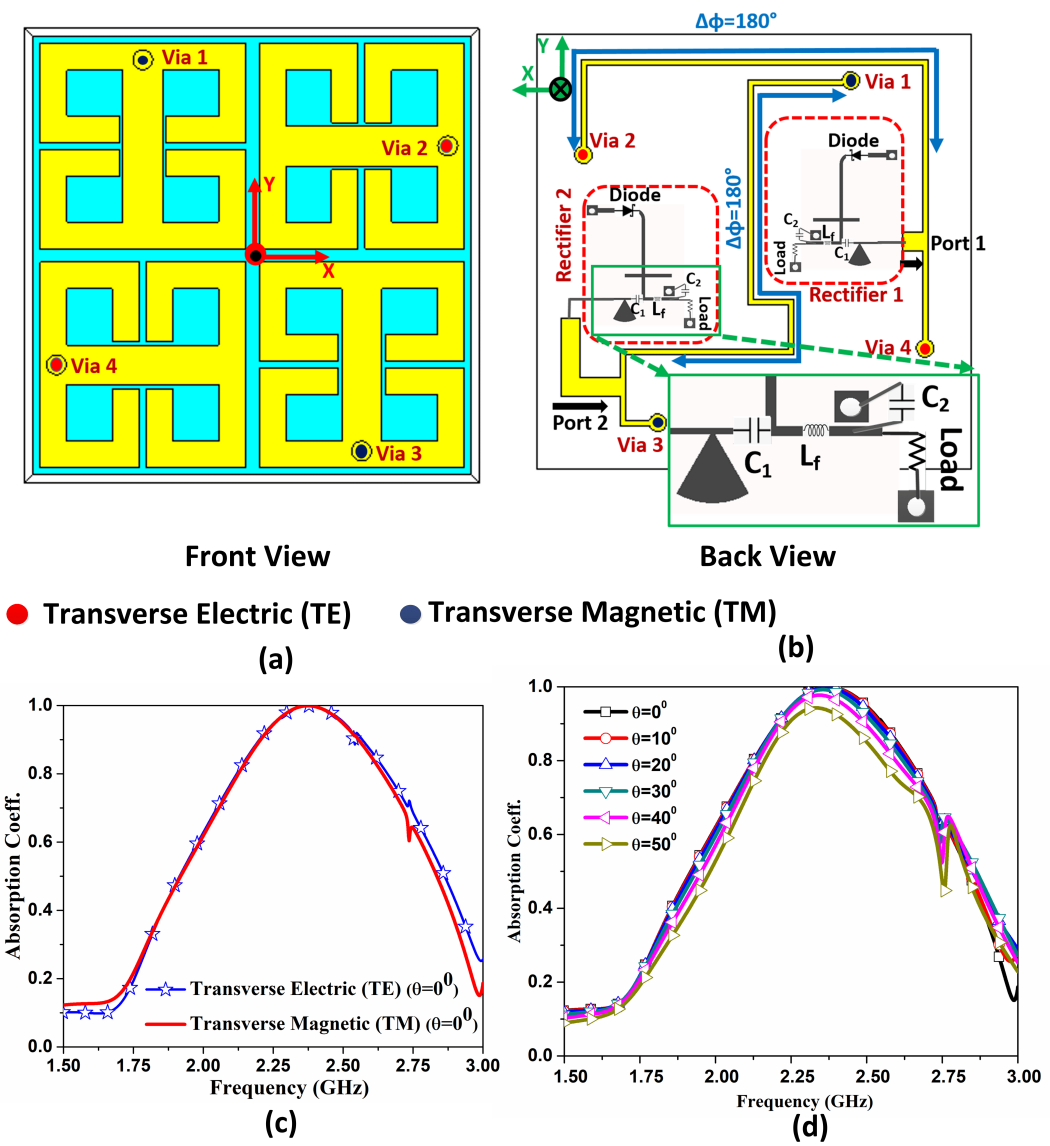
Representation of the supercell obtained from combination of 2 × 2 unit-cell (**a**) front view of the supercell (**b**) back view of the supercell with feed network (**c**) absorption for dual-polarised wave (**d**) the absorption for TM polarization when plane wave impinges on surface with different angle.

The designed supercell is shown in Figure 2a, here the cells are placed in a clockwise manner with 90° rotation to ensure absorption of both TE and TM plane waves. For better matching at different angles the supercell substrate thickness $H_{sub}$ was adjusted to 9.9 mm. Now, at the backside two symmetrically opposite cells are combined with 100 Ω microstrip lines with a 180° phase delay, (as shown in Figure 2b). The phase shift is necessary as the co-linearly polarised unit-cells are out-of phase due to the rotation within the supercell.

The combined power is fed into the rectifier with 50 Ω impedance in order to perfectly 100 Ω unit cells. The feed network is designed using RT/Duroid 5880 substrate with 0.5 mm thickness, identical to the rectifier. Here, for the brevity the two rectifiers are shown to be connected with the port 1 and 2 that correspond to TE and TM plane waves.

The designed supercell with the feed network at the backside was simulated using CST Microwave Studio. The finite element method (i.e., frequency domain solver) alongside with periodic boundary conditions (i.e., Floquet periodicity) around the supercell were applied. For the full electromagnetic analysis, the rectifier was modelled by its input impedance, using two lumped resistors of 50 Ω in our model. The excitation signal was a plane wave (periodic port), which impinges the supercell normally or by an angle, with a specific polarization. The maximum element size was always less than λ/7, where λ is the wavelength which corresponds to the design frequency (i.e., 2.45 GHz). The resultant absorption for the two polarization is presented in Figure 2c. This represents a very efficient operation of the supercell as the absorption efficiency is obtained as 98%. The supercell also demonstrates excellent performance with respect to the angle of incidence (i.e., polar angle θ), which is shown in Figure 2d. It can be noted here that the absorption is almost constant for the variation of incident wave angle, resulting in a wide-band and polarization insensitive MH. The sharp dip at 2.75 GHz is associated with parasitic resonance of the supercell and does not affect the performance of the MH at the design frequency.

**Figure 3:**
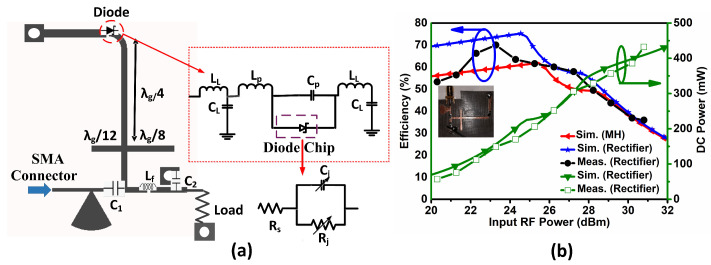
Rectifier description (**a**) the circuit diagram for the designed rectifier circuit with equivalent circuit model including leads of the diode in the inset, here the optimized lumped component values are $C_{1}$ = 2 pF, $C_{2}$ = 47 pF and $L_{f}$ = 55 nH; (**b**) the RF to DC conversion efficiency obtained at the optimized load.

The proposed topology for the rectifier is shown in Figure 3a, here the rectifier consists of a single diode in shunt configuration. A commercially available diode (Infineon BAT-68) was used for the rectifier circuit. Its spice model parameters are shown in the Table 2. To achieve better accuracy in the equivalent circuit model of the diode, the parasitic components ($C_{p}$ and $L_{p}$) and lead frame and the bond wires ($C_{L}$ and $L_{L}$) [35] are also considered, see the inset of Figure 3a.

For the high-power application our main aim is to choose a diode with quite good breakdown voltage at the cost of forward voltage. The forward voltage for the chosen diode ranges between 340 to 500 mV [36]. The breakdown voltage $V_{br}$ was obtained via an I-V characteristics measurement using Agilent’s Semiconductor Device Analyzer B1500A. The measured value was found to be 16 V, while in the datasheet it is given as 9 V. In our modeling, the measured value of the breakdown voltage was used. $R_{s}$ and $I_{s}$ are the internal sheet resistance and reverse saturation current, respectively, for the diode [36]. The nonlinearity of the diodes usually come from the nonlinear junction resistance $R_{j}$ and nonlinear junction capacitance $C_{j}$, where $C_{j0}$ is the zero bias junction capacitance [37]. It is worth remarking that the value of $R_{j}$ is approximately zero in the “ON” state and infinite in the “OFF” state of the diode.

The rectifier was designed and optimized with a matching network, which is a radial stub, in order to transfer maximum power from the feed port 1 and 2 of the front end of the metasurface. The prototype was fabricated on 0.5 mm thick RT/Duroid 5880 substrate. The structure of the rectifier is optimised for 20 to 30 dBm RF power at an operating frequency of 2.45 GHz. Extensive parametric studies showed that the optimal load value is 70 Ω, which is used in subsequent calculations.

Next, the measurement of the circuit has been performed for the verification of the topology. A comparison with the simulated results is shown in Figure 3b. The peak RF-to-dc conversion efficiency obtained was 70% in simulation and 68% from the measurement.

Afterwards, the metasurface unitcell was simulated with the measured rectifier results. For this, the simulated *S*-parameters for the unit cell of the metasurface were extracted from the CST Microwave Studio model [34]. The results of the calculated efficiency for the single port feed network in combination with one rectifier are given in Figure 3b. The obtained results demonstrate efficient operation of the proposed topology, especially for 22–26 dBm power range.

### 2.2. Direction of Arrival Estimation Using Metasurfaces

**Figure 4:**
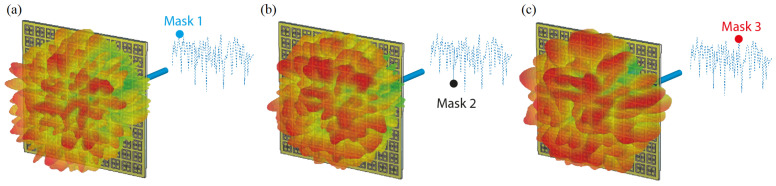
Spatio-temporally varying radiation patterns by the wave-chaotic metasurface antenna. The radiation patterns are demonstrated for the first three mask configurations (**a**) mask 1 (**b**) mask 2 (**c**) mask 3. The measured signal at the compressed channel is also shown with the corresponding mask configuration number highlighted.

As well as synthesizing an absorption response for incoming waves, a metasurface can also be designed to operate in transmission mode to radiate tailored field patterns. As a transmission metasurface, a particularly interesting application can be considered in the context of compressive sensing facilitated by wave-chaotic metasurface antennas. To achieve this, a complementary version of the SRR unit-cell structure presented in Section 2.1 can be used to design a single-pixel wave-chaotic metasurface aperture to facilitate physical layer compression. This single-pixel detector scheme has recently received significant traction to synthesize wave-chaotic compressive sensing apertures at microwave and millimetre-wave frequencies [5,38,39]. A significant advantage of wave-chaotic metasurface antennas is that they can sample the scene information in an *indirect* manner, eliminating the need for raster scanning. This is achieved by encoding the backscatter measurements onto a set of spatio-temporally varying quasi-random field patterns radiated by wave-chaotic metasurface antennas. The compressive sensing concept facilitated by single-pixel metasurface antennas can also find applications in channel-characterization as an enabling technology for localization and direction of arrival estimation (DoA) [40,41]. In this context, the spectrum of the far-field sources incident on the wave-chaotic metasurface aperture are compressed into a single channel and an estimate of this spectrum is recovered using the transfer function of the wave-chaotic metasurface antenna. In Figure 4, we present a wave-chaotic metasurface antenna consisting of an array of unit-cells, complementary to those developed for the energy harvesting surface presented in Section 2.1. The unit-cells were printed on a dielectric substrate, Rogers 4003 ($\epsilon_{r}=3.38$). The metasurface is fed in the aperture centre in the transverse plane (xy-plane) with a cylindrical guided-mode inside the substrate exciting the metasurface. The operating frequency was selected to be 10 GHz and the complementary SRR unit-cells are designed accordingly to resonate at 10 GHz frequency.

**Figure 5:**
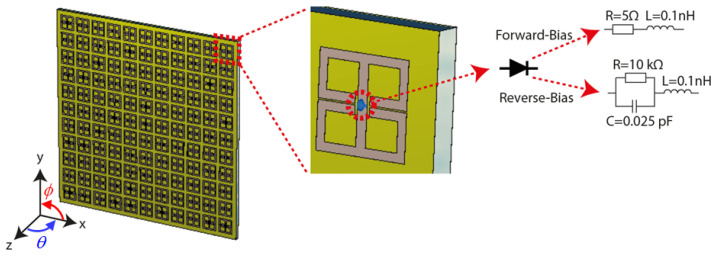
Dynamic modulation of the unit-cell topology using PIN diodes. The equivalent circuit states of the PIN diodes are also shown.

The electrical size of the metasurface in Figure 4 was 15λ × 15λ, where λ denotes the wavelength at the operating frequency 10 GHz. It should be noted that, despite the large electrical size, the wave-chaotic metasurface exhibits a single channel for data acquisition facilitated by the hardware layer compression. As can be seen in Figure 4, the wave-chaotic metasurface radiates quasi-random radiation patterns by means of actively modulating the unit-cells across the metasurface. In this scheme, each metasurface configuration with a randomized *on*/*off* element distribution constitutes a *mask*, with each mask radiating a different, quasi-random pattern. For the presented technique, this modulation is achieved by loading the unit-cells with PIN diodes [42]. The role of the PIN diodes is to dynamically control the radiation characteristics of the unit-cells by tuning the unit-cells *on*/*off*. In unit-cell *on* state, the element couples to the guided-mode and radiates into free space. This is achieved by reverse-biasing the PIN diodes short-circuiting the unit-cell as depicted in Figure 5. In unit-cell *off* state, the element does not couple to the guided-mode, and hence, does not radiate. The *off* state modulation of the unit-cell can be realized by forward-biasing the PIN diodes as depicted in Figure 5. In this work, the selected PIN diodes to control the radiation response of the unit-cells is MACOM MADP-000907-14020W [5], with its circuit diagram for forward and reverse bias configurations being shown in Figure 5.

**Figure 6:**
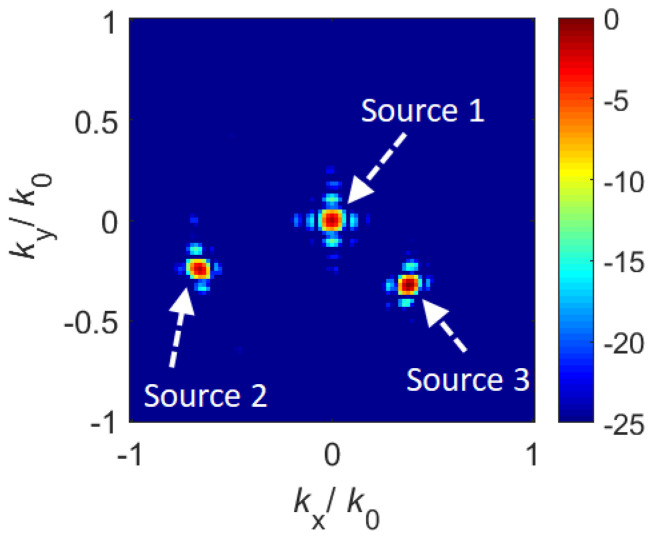
Reconstructed DoA pattern for the incoming far-field sources encoded by the wave-chaotic metasurface antenna. Colorbar: in dB scaling.

In Figure 6, we present the application of the developed coded metasurface aperture for DoA estimation. For this study, we consider an arbitrarily selected number of far-field sources incident on the aperture at ($\theta_{1}=0{}^{\circ},\phi_{1}=0{}^{\circ}$), ($\theta_{2}=-45{}^{\circ},\phi_{2}=20{}^{\circ}$) and ($\theta_{3}=30{}^{\circ},\phi_{3}=-40{}^{\circ}$), respectively. Whereas the DoA estimation of 3 different far-field sources is studied in this article, we note that the number of far-field sources can be varied without loss of generality for the presented technique.

For the studied DoA scenario, the wave-chaotic metasurface antenna synthesizes 101 wave-chaotic modes radiated by means of sweeping through 101 different aperture mask configurations. The compressed signal measured at the channel of the wave-chaotic metasurface is the superposition of the individual contributions of the far-field sources. From the compressed measurements of the channel, *g*, an estimate of the far-field sources on the metasurface aperture, $f_{est}$, can be retrieved as follows:

(3) $$f_{est}=H^{+}g$$

In Equation (3), *H* denotes the transfer function of the wave-chaotic metasurface antenna. Equation (3) is a single-shot matched-filtering algorithm and can be applied in real-time [43]. Following the estimation of the far-field sources on the antenna aperture, the DoA estimation can be achieved by means of a simple Fourier transform operation applied to $f_{est}$. In Figure 6, we present the reconstructed DoA patterns whereas a quantitative error analysis of the reconstructed DoA values is presented in Table 3.

As shown in Table 3, the retrieved DoA values exhibit good agreement with the actual DoA information.

### 2.3. Fundamental Building Block of Intelligent Reflecting Surfaces

**Figure 7:**
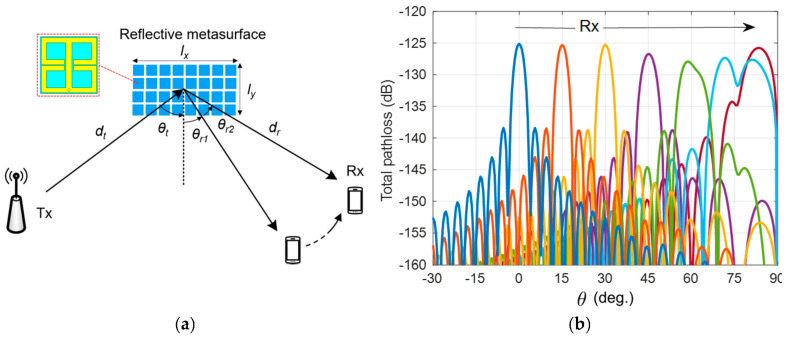
(**a**) Pathloss system model with non-ideal reflective metasurface developed using an array of unit-cells, used as an IRS, (**b**) Total path loss incurred by received signal at the receiver device when it is moved along polar angle (θ).

The metamaterial concept also plays a fundamental role in building reflection based surfaces, particularly in the context of intelligent reflecting surfaces (IRS) [10,11,44]. An IRS consisting of ideal unit-cells is preferred (and often assumed) to have a uniform gain radiation mask within a coverage area along polar and azimuth (i.e., ϕ) directions [45,46,47]. This is not the case when practical unit-cells like the one shown in Figure 5 are used to build a reflective metasurface used as an IRS. To explain this, let us look at a simulation setup given in Figure 7 in which a pathloss system model is implied to record received signal profile when it is served by a non-ideal (practical) IRS. We considered a metasurface having a length of $l_{x}=15\lambda$ along the *x*-axis and $l_{y}=15\lambda$ along the *y*-axis. The total path loss at the receiver when it’s distance from the metasurface is $d_{r}$ and the distance between the transmitter and the metasurface is $d_{t}$ can be written as:

(4) $$\mathrm{Total}\mathrm{Pathloss}=\frac{G_{t}\times G_{m}\left(\theta \right)\times G_{r}}{4\pi^{2}}{\left(\frac{l_{x}l_{y}}{d_{t}d_{r}}\right)}^{2}\cos^{2}\left(\theta_{i}\right){\left(\frac{\sin\left(\frac{\pi l_{x}}{\lambda }\left(\sin\left(\theta \right)-\sin\left(\theta_{r}\right)\right)\right)}{\frac{\pi l_{x}}{\lambda }\left(\sin\left(\theta \right)-\sin\left(\theta_{r}\right)\right)}\right)}^{2}$$

When $G_{t}$ and $G_{r}$ are the transmitter and receiver antenna gains [47,48], $G_{m}\left(\theta \right)$ is the metasurface reflective gain pattern along the polar angle θ, while $\theta_{i}$ and $\theta_{r}$ are the transmitter and receiver positions relative to the metasurface’s broadside direction. For the sake of simplicity in analysis, consider uniform gains of the transmitter and receiver antennas $G_{t}=G_{r}=3$ dBi, and uniform distance between transmitter and metasurface ($d_{t}$), and a uniform distance between metasurface and the receiver ($d_{r}$), i.e., $d_{t}=d_{r}=30$ m. We simulated a receiver moving from θ=0° to θ=90° as shown in Figure 7, while the metasurface was separately configured to serve the receiver at intervals 15° apart within the polar angle range of θ=0° to θ=90°. The simulated results are presented in Figure 7. It is clear that the total pathloss profile is close to ideal (and also predictable) when the receiver is located at the IRS broadside, i.e., 0°, but as the receiver moves away from the broadside along the polar direction, the pathloss profile deviates from ideal, and becomes almost unpredictable after a specific polar angle (in the given example, after 60°). Thus, contrary to popular belief, the results in Figure 7 show that the service quality of an IRS-aided communication system relies significantly on equalized and consistent radiating fields from the reflective metasurfaces. This makes designing and testing of non-ideal unit-cells a research priority if we wish to achieve efficient IRS design, which can facilitate high data rate communication.

## 3. Discussion

The use of metamaterial absorbing surfaces as energy harvesting panels in wireless power transfer applications provides an exciting alternative to existing energy harvesting arrays based on rectenna systems [49,50,51,52]. Specifically, MH have the potential for high absorption of electromagnetic radiation, compact size due to the subwavelength unit-cell dimensions, possibility for low cost fabrication using traditional milling but also inkjet printing fabrication, and conformal implementation using flexible substrates [20]. Furthermore, they can be tuned or even adaptively tuned to respond to different frequency bands of interest. The use of metamaterials enables the realization of “*smart surfaces*” capable of absorbing electromagnetic radiation for energy recycling.

In this work, we initially presented a new polarisation insensitive and wide-angle MH, based on the geometry which was proposed in [20]. The new MH has been designed to operate for a WPT application at 2.45 GHz and for high power input (20–30 dBm). The measured peak RF-to-dc efficiency was 68% for 23 dBm RF power input, which yields dc output power of 150 mW. The maximum dc power of 440 mW was measured at input power of 30 dBm. The MH demonstrates very efficient operation across the 22–26 dBm range of input power, thus allowing designing larger MH for medium to high-power ranges.

It is interesting to note that by employing the supercell approach we were able to keep the wide-angle operation. Any further optimisation of the structure, such as wideband or multi-band operation, can be achieved at the unit cell level. The same applies to the rectification circuitry as the power-combining mechanism designed for the unit cell in this paper will be easy to adapt for different topologies of the rectifier circuit. The wide-angle and dual-polarisation operation will also enable efficient WPT operation at different angles between the source and the object being charged, as the alignment condition can be relaxed. This feature is of particular importance for remote charging of moving objects, such as UAVs.

For the wave-chaotic metasurface-based compressive DoA estimation technique presented in Section 2.2, the reconfiguration mechanism of the metasurface aperture plays a key role. Because compressive DoA estimation relies on spatio-temporally varying the aperture radiated fields to synthesize the wave-chaotic modes and probe the sources incident on the aperture, methods to achieve this wave-chaotic operation deserve some discussion. To achieve the synthesis of the wave-chaotic modes, two modulation techniques can be used; frequency-diversity [38] and dynamic aperture [5,53]. The compressive DoA estimation framework presented in Section 2.2 relies on the dynamic aperture principle, leveraging PIN diodes to control the radiation response of the unit-cells and create random mask distributions across the metasurface aperture by means of a binary modulation scheme (*on/off*) [5]. In contrast, the frequency-diversity technique leverages a passive, static metasurface architecture, and relies on a frequency-sweep to synthesize the wave-chaotic modes. To facilitate the frequency-diversity technique, two methods can be used; in the first method, the unit-cells across the metasurface aperture can be distributed randomly in terms of their geometrical features, suggesting that the unit-cells forming the metasurface layer have randomly distributed resonance frequencies [54]. In this method, as the frequency is swept, it is ensured that a random subset of unit-cells will radiate at each sampling frequency within the sweep, generating a set of spatio-temporally varying modes as a function of frequency. In the second method, the diversity mechanism can be originated by the guided-mode, exciting the metasurface layer by means of using a cavity-backed feeding architecture behind the metasurface [55]. As the operating frequency is swept, the cavity-mode inside the feeding structure undergoes spatial variation, thereby resulting in a different radiation pattern when exciting the metasurface. Regardless of the type of method used to realize the frequency-diverse operation, it is important to emphasize that the frequency-diverse operation requires that a certain frequency band be swept. Because the frequency-diversity technique requires a frequency sweep to synthesize the wave-chaotic radiation patterns, it brings several disadvantages compared to the dynamic aperture concept. First, the necessity to do a frequency-sweep increases the complexity of the RF signal generation and processing units. Second, for a given antenna quality-factor (or Q-factor), reducing the correlation between the wave-chaotic modes requires that the separation between the frequency sampling points be increased, directly translating into wider frequency bandwidths. Third, and finally, in order for the frequency-diversity technique to work for compressive DoA estimation, the variation in the spectrum of far-field sources to be detected should be negligible as the frequency is swept to synthesize the wave-chaotic modes from the metasurface aperture [40,41]. To ensure this, the frequency bandwidth should be kept minimal, which is in direct conflict with the second item listed above in terms of mode diversity. As a result of these factors, for the studies presented in Section 2.2, we leveraged the dynamic aperture principle operating at a single frequency, 10 GHz, with the wave-chaotic modes being synthesized by actively modulating the unit-cells across the metasurface aperture using PIN diodes.

**Figure 8:**
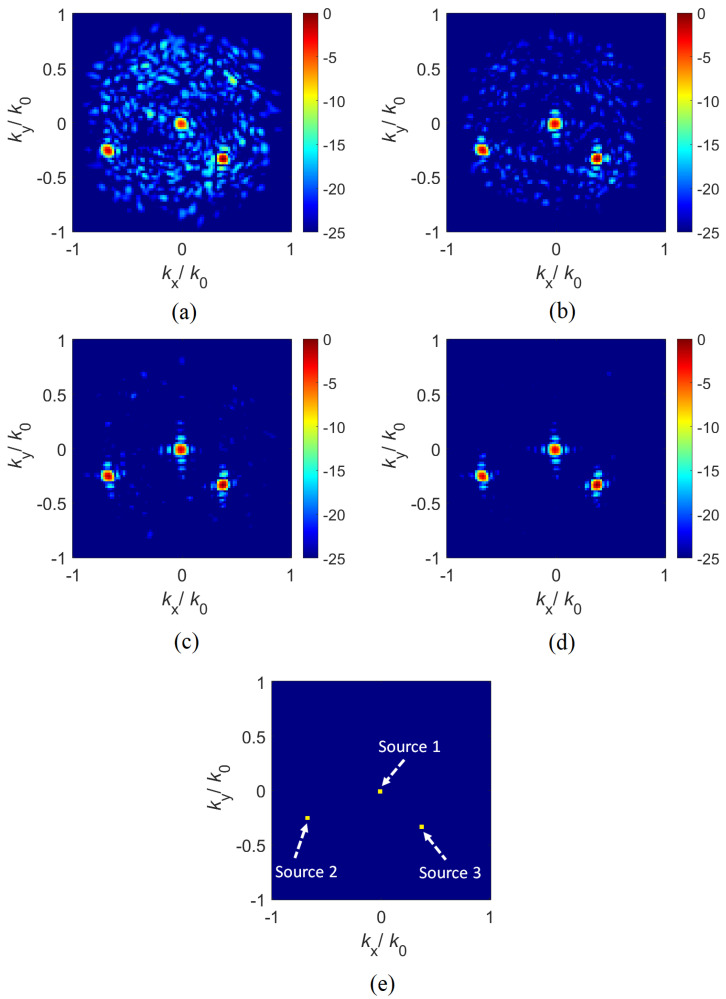
Reconstructed DoA patterns as a function of varying SNR levels (**a**) SNR=0 dB (**b**) SNR=5 dB (**c**) SNR=10 dB and (**d**) SNR=15 dB. Ground truth is shown in (**e**). Colorbar for (**a**–**d**): in dB scaling.

Another aspect for the compressive DoA estimation concept is the robustness of this technique to system noise, which is an important consideration for practical applications. For the studies shown in Section 2.2, the data measured at the compressed channel was assumed to be noiseless. Whereas such an assumption can be useful to analyze the fidelity of the reconstructed DoA patterns under ideal conditions as a best case scenario, in reality, the acquired compressed data would exhibit a finite signal-to-noise ratio (SNR) level caused by various system noise factors present in measurements. To this end, in order to present how the compressive DoA estimation concept would perform under various SNR conditions, in Figure 8, we present the reconstructed DoA patterns for various SNR values across the range of 0–15 dB at 5 dB intervals. For this analysis, the studied DoA estimation scenario is identical to the noiseless scenario originally studied in Figure 6 except in this case, complex-valued Gaussian distributed noise is added to the data in a manner similar to that described in [43]. The reconstructed DoA patterns as a function of varying SNR levels are shown in Figure 8.

A careful investigation of Figure 8 reveals that the compressive DoA estimation technique can recover the DoA information even where the SNR=0 dB. This is testament to the ability of the proposed compressive DoA estimation technique to work under relatively low SNR conditions. Moreover, investigation around non-ideal IRS in Figure 7 reveals that the research around unit-cell of reflective type metasurface is an interesting research problem, emphasizing the need of resonant electric based metamaterials for electromagnetic wave manipulation.

## 4. Conclusions

We presented ELC-based metasurface apertures for EM wave manipulation within the microwave frequency spectrum. In this context, a variety of metasurface apertures were developed and implemented as an enabling technology for emerging wireless applications including efficient wireless power transfer at distance and compressive DoA estimation for wireless sensing. Geometry was tested through full-wave electromagnetic analysis and measurements and good agreement between simulated and measurement results was observed: the proposed structure presents high RF-to-dc efficiency and the retrieved DoA information was in excellent agreement with the original DoA information of multiple far-field sources. Specifically, as a RF energy harvester, the proposed metasurface structure presents high absorption efficiency, i.e., 98% and high measured RF-to-dc efficiency, i.e., 68%, and thus, it can be utilised in WPT applications. As an enabling technique for compressive DoA estimation, a single-channel wave-chaotic metasurface antenna was developed and presented to retrieve the DoA information of multiple far-field sources. It was demonstrated, qualitatively and quantitatively, that the retrieved DoA information was in excellent agreement with the original DoA information. Finally, please note that, although the frequency targeted in this work was in the microwave region of the spectrum, the presented metasurface design can readily be scaled to higher operating frequencies.

## Figures and Tables

**Table 1:** The unit-cell dimensions (mm) for the WPT metasurface.

Parameter	a	c	d	e	g	s	w	$H_{\mathrm{sub}}$
**Value**	16.31	7	3	5.6	0.5	0.5	2	5.5

**Table 2:** Rectifier diode equivalent circuit values.

Parameter	$R_{s}$	$I_{s}$	$C_{j0}$	$V_{\mathrm{br}}$
**Value**	1.49 Ω	9 nA	0.786 pF	16 V

**Table 3:** Comparison between the original DoA and estimated DoA values.

	Original DoA	Estimated DoA
**Source 1**	$\theta_{1}=0{}^{\circ},\phi_{1}=0{}^{\circ}$	$\theta_{1}=0{}^{\circ},\phi_{1}=0{}^{\circ}$
**Source 2**	$\theta_{2}=-45{}^{\circ},\phi_{2}=20{}^{\circ}$	$\theta_{2}=-44.7{}^{\circ},\phi_{2}=19.9{}^{\circ}$
**Source 3**	$\theta_{3}=30{}^{\circ},\phi_{3}=-40{}^{\circ}$	$\theta_{3}=29.8{}^{\circ},\phi_{3}=-40.1{}^{\circ}$

## Data Availability

Not applicable.
